# Accuracy of behavioral health variables in Oregon national violent death reporting system data: a linked cohort study

**DOI:** 10.1186/s40621-022-00393-7

**Published:** 2022-09-13

**Authors:** Kathleen F. Carlson, Tess A. Gilbert, Susan DeFrancesco, Dagan A. Wright, Xun Shen, Lawrence J. Cook

**Affiliations:** 1 VA HSR&D Center to Improve Veteran Involvement in Care (CIVIC), VA Portland Health Care System (R&D 66), 3715 SW U.S. Veterans Hospital Road, Portland, OR 97239 USA; 2 Oregon Health & Science University, Portland State University School of Public Health, Portland, OR USA; 3 Injury and Violence Prevention Section, Public Health Division, Oregon Health Authority, Portland, OR USA; 4 Division of Critical Care, Department of Pediatrics, University of Utah, Salt Lake City, UT USA

**Keywords:** Assault, Firearm, Homicide, Suicide, Oregon violent death reporting system, Violence

## Abstract

**Background:**

The National Violent Death Reporting System (NVDRS) collects data on the circumstances of violent deaths, and all firearm-related deaths, across states and territories in the USA. This surveillance system is critical to understanding patterns and risk factors for these fatalities, thereby informing targets for prevention. NVDRS variables include behavioral health conditions among decedents, but the validity of the reported behavioral health data is unknown. Using Department of Veterans Affairs (VA) healthcare records as a criterion standard, we examined the accuracy of NVDRS-reported behavioral health variables for veteran decedents in a sample state (Oregon) between 2003 and 2017.

**Methods:**

We linked Oregon NVDRS data to VA healthcare data to identify veteran decedents who used VA services within two years of death. Veterans’ VA diagnoses within this time frame, including depression, post-traumatic stress disorder (PTSD), anxiety, and substance use disorders, were compared to behavioral health variables identified in the Oregon NVDRS. Concordance, sensitivity, and correlates of sensitivity were examined over time and by decedent characteristics.

**Results:**

We identified 791 VA-using veterans with violent and/or firearm-related fatal injuries documented in the Oregon NVDRS between 2003 and 2017. In this cohort, the Oregon NVDRS accurately identified only 49% of decedents who were diagnosed with depression, 45% of those diagnosed with PTSD, and 17% of those diagnosed with anxiety by the VA. Among 211 veterans diagnosed by the VA with a substance use disorder, the Oregon NVDRS coded only 56% as having a substance use problem. In general, the sensitivity of behavioral health variables in the Oregon NVDRS remained the same or decreased over the study period; however, the sensitivity of PTSD diagnoses increased from 21% in 2003–2005 to 54% in 2015–2017. Sensitivity varied by some decedent characteristics, but not consistently across behavioral health variables.

**Conclusions:**

NVDRS data from one state missed more than half of behavioral health diagnoses among VA-using veterans who died from violence or from firearm injuries. This suggests that reports of behavioral health conditions among decedents nationally may be severely undercounted. Efforts to improve validity of these variables in state NVDRS data are needed.

## Introduction

In 2020, approximately 71,335 people died from intentional injuries in the United States (US); nearly 46,000 died by suicide and more than 25,000 by homicide (US Centers for Disease Control and Prevention 2022). Firearms were the most common source of violent injury, used in approximately half of all suicides and three-quarters of all homicides, and also accounted for a considerable number of unintentional injury deaths (US Centers for Disease Control and Prevention 2022). Military veterans are at heightened risk of fatal injury following military deployments and, in particular, experience high rates of suicide, especially firearm suicide, compared to non-veterans (Kang and Bullman 2001; Knapik et al. 2009; Reger et al. 2018; US Department of Veterans Affairs 2021). The systematic examination of the circumstances of violent death in the US is critically important to informing public health-based prevention programs that will save lives, especially those of US military veterans.

The National Violent Death Reporting System (NVDRS), a surveillance system funded by the US Centers for Disease Control and Prevention (CDC) to monitor and gather information about violent deaths in the US, links death certificate data to coroner and medical examiner reports, law enforcement reports, and, where appropriate, toxicology reports, to provide detailed information about the circumstances and characteristics associated with these injury fatalities (US Centers for Disease Control and Prevention 2021). The NVDRS began data collection in 2003 and included just six participating states (Maryland, Massachusetts, New Jersey, Oregon, South Carolina, and Virginia; Steenkamp et al. 2006). Since then, states were added incrementally and, in 2018, the NVDRS expanded data collection to include all 50 states, the District of Columbia, and Puerto Rico (Nazarov et al. 2019). Deaths captured in the NVDRS include suicides, homicides, deaths due to legal intervention (excluding executions), deaths of undetermined intent that might have been due to violence, deaths due to terrorism (excluding acts of war), and all deaths caused by firearm discharges, regardless of their intent (i.e., intentional or unintentional; Steenkamp et al. 2006; Blair et al. 2016; Nazarov et al. 2019).

Over the years it has existed, the NVDRS has systematically provided detailed data on the circumstances of violent deaths among US residents. Notably, the NVDRS has been used to better understand fatalities among military veterans, often by comparing deaths among veteran decedents to those of non-veteran decedents (Safe States Alliance 2018). A prevention target that is frequently the focus for veteran populations—especially given higher rates of suicide—is behavioral health; indeed, a number of NVDRS reports have shown that higher numbers of military veterans had depressed mood or other behavioral health disorders and diagnoses, including depression, post-traumatic stress disorder (PTSD), or substance abuse issues, than non-veteran decedents (Kaplan et al. 2009, 2012; Shen and Millet 2014; Logan et al. 2016; Safe States Alliance 2018). NVDRS data are not clinical in nature, and the absence of a behavioral health indicator does not necessarily represent medically confirmed absence of the respective behavioral health condition; however, the patterns observed in these data can inform national strategies to prevent suicide and other causes of premature death, as evidenced by the US Department of Veterans Affairs (VA) National Strategy for Preventing Veteran Suicide (Department of Veterans Affairs 2021).

Behavioral health conditions are important targets for intervention among not just veterans, but all individuals at risk for violent death and, as such, the accuracy of these variables in the NVDRS is critically important. However, little is known about the validity of NVDRS behavioral health-related data, particularly in contrast to decedents’ actual healthcare records. The purpose of this analysis was to: (1) Estimate the accuracy of NVDRS-reported behavioral health data for veteran decedents in a single state (Oregon); and (2) Examine patterns of accuracy over time and by decedents’ demographic, military service, and death-related characteristics.

## Methods

We conducted a secondary analysis of data from a research project examining fatal and nonfatal firearm injuries among veterans in Oregon. The conduct of the research project, including this sub-study, was approved by the Oregon Health Authority, Public Health Division, and the joint Department of Veterans Affairs (VA) Portland Health Care System-Oregon Health and Science University Institutional Review Boards.

### Data sources

We utilized administrative data from the VA Corporate Data Warehouse (US Department of Veterans Affairs 2022) and the VA/Department of Defense Identity Repository (US Department of Veterans Affairs 2009). We obtained Oregon NVDRS data from the Oregon Public Health Division Injury and Violence Prevention Program (Oregon Health Authority 2022), which oversees data collection for the state. VA administrative data were initially restricted to veterans who had at least one visit to an Oregon VA healthcare facility between 2001 and 2017 and whose most recent residential address was in Oregon or Washington. Oregon NVDRS records from 2003 through 2017 were then linked to the VA administrative data to identify all veteran decedents who were enrolled in the VA healthcare system. Procedures for linking these databases were governed by a data use agreement negotiated between the VA and the Oregon Public Health Division.

We used probabilistic linkage, as implemented in LinkSolv software version 9.0 (Morrisville, NY), to link VA and Oregon NVDRS data. Probabilistic linkage is a method used when a unique identifying key is not available across the databases to be linked (for example, Social Security Numbers were available in VA data but not in Oregon NVDRS data). This method uses properties of variables that are common across the databases to estimate the probability that a pair of records refer to the same person or event and should be linked (Fellegi and Sunter 1969; Jaro 1995; Cook et al. 2001). Variables used to link VA and Oregon NVDRS data for this study included full names, dates of birth, sex, and addresses (city and zip code); other variables such as cause of death, circumstances of death, or behavioral health variables were not utilized in this linkage process. A pair of records was required to achieve a probability of at least 0.8 to be considered a true match. All matched records were retained regardless of the completeness of behavioral health or descriptive variables in the corresponding NVDRS and VA data.

**Fig. 1:**
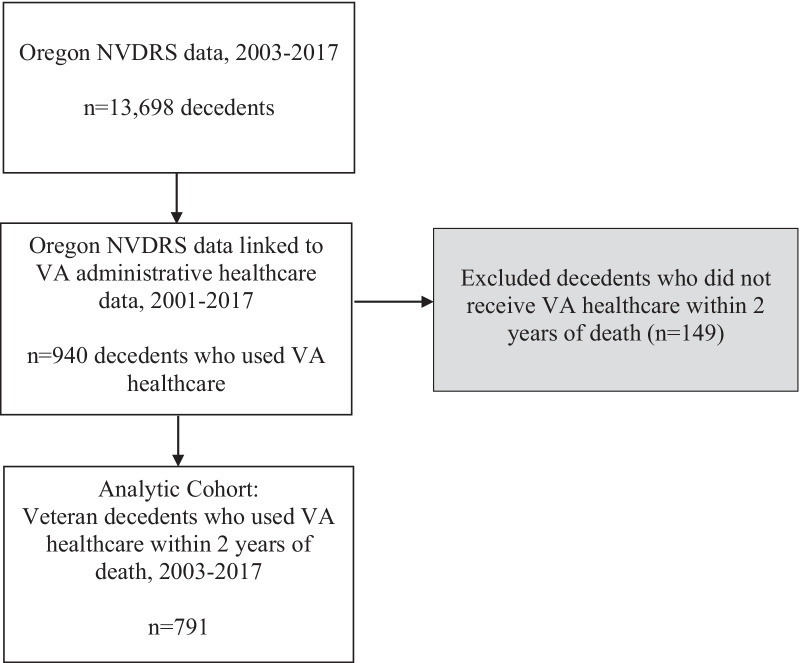
Flow of Participants from Source Population to Analytic Cohort

This linkage resulted in a dataset of veteran decedents who had used VA healthcare prior to death. To capture veterans’ behavioral health status during the period most proximal to their death, we restricted the dataset to those who had received VA healthcare within two years of dying. The numbers of veterans retained in each step from the source population (*n* = 13,698 veteran and non-veteran decedents in the Oregon NVDRS, 2003–2017) into this final analytic cohort (*n* = 791 veterans who used VA healthcare within two years of death) are depicted in Fig. 1.

### Behavioral health variables

NVDRS data collection includes any mental health diagnoses listed in the Diagnostic and Statistical Manual of Mental Disorders, Fifth Edition, as described in the NVDRS coding manual (US Centers for Disease Control and Prevention 2021). This information, from medical examiner reports on death scene investigations and/or incident reports of local law enforcement, is entered by NVDRS abstractors using a drop-down menu of the most common diagnoses and a field for “other” in which they can code less common diagnoses. The most common diagnoses include depression/dysthymia, PTSD, and anxiety disorder (US Centers for Disease Control and Prevention 2021); we focused on these three disorders given their relatively high prevalence in veterans (Trivedi et al. 2015). (Although serious mental illnesses such as bipolar disorder and schizophrenia are also important concerns among the veteran population, the prevalence of these conditions among decedents was comparatively low and therefore not independently examined in this investigation.) The entries for these diagnoses are intended to be made in the NVDRS system only if abstractors find evidence of an actual clinical diagnosis for a respective disorder in medical examiner and/or law enforcement reports; still, these entries are best understood as proxies for diagnosis.

Additional variables in the NVDRS are not dependent on evidence of a clinical diagnosis but are intended to be coded with any indication from the collection of decedents’ records. These include alcohol or other substance abuse problems, any indication of a mental health problem, or current depressed mood (perceived by the decedent themselves or by others; US Centers for Disease Control and Prevention 2021). The first two of these variables are also intended to be coded if decedents had alcohol or drugs (amphetamines, cocaine, marijuana, or opioids) in their systems at the time of death (coded as alcohol or substance abuse problems) or current prescriptions for antidepressants or other psychiatric medications (coded as mental health problem; US Centers for Disease Control and Prevention 2021).

Diagnoses in VA healthcare data were identified using International Classification of Diseases, 9th and 10th revisions, Clinical Modification diagnosis codes (ICD-9-CM and ICD-10-CM; US Department of Health and Human Services 2008; US National Center for Health Statistics 2022). To identify equivalent diagnoses between ICD-9-CM and ICD-10-CM coding schema, we used the Centers for Medicare & Medicaid Services General Equivalency Mapping tables with a forward–backward method (US Centers for Medicare & Medicaid Services 2021). We coded veterans as having diagnoses of interest (depression, PTSD, anxiety disorders, substance use disorders, or any behavioral health disorder) if they had one or more of the respective diagnosis codes assigned during a VA inpatient stay, or two or more codes assigned during VA outpatient visits, within the two-year period prior to their death.

For all variables of focus for this analysis, we present the data fields we used from the Oregon NVDRS data, and the ICD-9-CM and ICD-10-CM codes used to develop indicators of the corresponding behavioral health diagnoses from VA data, in Table 1. Of note, the variables representing “any behavioral health disorder” included those of primary focus (depression, PTSD, anxiety disorders, substance use disorders) as well as serious mental illnesses such as bipolar disorder and schizophrenia.

**Table 1:** Oregon NVDRS and corresponding VA Healthcare System behavioral health variables compared in analyses

	Oregon NVDRS^a^	VA healthcare system^b^	
Variable	Data field and required value	ICD-9-CM codes	ICD-10-CM codes
Depression/ Dysthymia Diagnosis	CME/LE_MentalHealthDiagnosis1 = Depression, *or* CME/LE_MentalHealthDiagnosis2 = Depression, *or* CME/LE_MentalHealthDiagnosisOther = Depression	296.20–296.26, 296.30–296.36, 300.4, 311	F320-F325, F329-F3342, F339, F341
PTSD Diagnosis	CME/LE_MentalHealthDiagnosis1 = PTSD, *or* CME/LE_MentalHealthDiagnosis2 = PTSD, *or* CME/LE_MentalHealthDiagnosisOther = PTSD	309.81	F4310, F4311, F4312
Anxiety Diagnosis	CME/LE_MentalHealthDiagnosis1 = Anxiety, *or* CME/LE_MentalHealthDiagnosis2 = Anxiety, *or* CME/LE_MentalHealthDiagnosisOther = Anxiety	300.00, 300.02, 300.09, 300.20, 300.22–300.23, 300.29, 300.3	F4000, F4002, F4010-F4011, F40210, F40218, F40220, F40228, F40230-F40233, F40240-F40243, F40248, F40290-F40291, F40298, F408-F409, F411, F413, F418-F419, F42
Substance Abuse Problems/ Substance Use Disorder Diagnosis	CME/LE_AlcoholProblem = Yes, *or* CME/LE_SubstanceUseOther = Yes	291.0–291.5, 291.9, 292.0, 292.2, 292.9, 291.81–291.82, 291.89, 292.11–292.12, 292.81–292.85, 292.89, 303.00–303.03, 303.90–303.93, 305.00–305.03, 304.00–304.03, 304.70–304.73, 304.80–304.83, 305.50–305.53, 304.10–304.13, 304.20–304.23, 304.30–304.33, 304.40–304.43, 304.50–304.53, 304.60–304.63, 304.90–304.93, 305.20–305.23, 305.30–305.33, 305.40–305.43, 305.60–305.63, 305.70–305.73, 305.80–305.83, 305.90–305.93, 648.30–648.34	F1010, F10120-F10121, F10129, F1014, F10150-F10151, F10159, F10180-F10182, F10188, F1019-F10221, F10229-F10232, F10239, F1024, F10250-F10251, F10259, F1026-F10282, F10288, F1029, F10920-F10921, F10929, F1094- F10951, F10959-F10982, F10988, F1099-F1110, F11120-F11122, F11129, F1114-F11151, F11159, F11181-F11182, F11188, F1119-F11222, F11229, F1123-F11251, F11259, F11281-F11282, F11288-F1129, F1190, F11920-F11922, F11929-F11951, F11959, F11981-F11982, F11988, F1199, F1210, F12120-F12122, F12129, F12150- F12151, F12159, F12180, F12188, F1219, F1220-F12222, F12229, F12250-F12251, F12259, F12280, F12288, F1229, F1290, F12920-F12922, F12929, F12950-F12951, F12959, F12980, F12988, F1299, F1310, F13120-F13121, F13129, F1314, F13150-F13151, F13159, F13180-F13182, F13188, F1319, F1320-F13221, F13229-F13232, F13239, F1324, F13250-F13251, F13259-F1327, F13280-F13282, F13288, F1329, F1390, F13920-F13921, F13929-F13932, F13939-F1394, F13950-F13951, F13959, F1396-F13982, F13988, F1399, F1410, F14120-F14122, F14129, F1414-F14151, F14159, F14180-F14182, F14188, F1419-F14222, F14229-F14251, F14259, F14280-F14282, F14288, F1429, F1490, F14920-F14922, F14929, F1494, F14950-F14951, F14959, F14980-F14982, F14988, F1499, F1510-F15122, F15129, F1514-F15151, F15159, F15180-F15182, F15188, F1519-F1521, F15220-F15222, F15229, F1523-F15251, F15259, F15280-F15282, F15288, F1529, F1590, F15920-F15922, F15929, F1593-F15951, F15959, F15980-F15982, F15988, F1599, F1610, F16120-F16122, F16129, F1614, F16150-F16151, F16159, F16180, F16183, F16188, F1619, F1620-F16221, F16229, F1624-F16251, F16259, F16280, F16283, F16288, F1629, F1690, F16920-F16921, F16929, F1694, F16950-F16951, F16959, F16980, F16983, F16988, F1699, F17203, F17208-F17209, F17213, F17218-F17219, F17223, F17228-F17229, F17293, F17298-F17299, F1810, F18120-F18121, F18129, F1814, F18150-F18151, F18159, F1817, F18180, F18188, F1819-F18221, F18229, F1824, F18250-F18251, F18259, F1827, F18280, F18288, F1829, F1890, F18920-F18921, F18929, F1894, F18950-F18951, F18959, F1897, F18980, F18988, F1899, F1910, F19120-F19122, F19129, F1914, F19150-F19151, F19159-F1916, F1917, F19180-F19182, F19188, F1919-F19222, F19229-F19232, F19239, F1924, F19250-F19251, F19259-F1927, F19280-F19282, F19288, F1929, F1990, F19920-F19922, F19929-F19932, F19939, F1994-F19951, F19959-F1997, F19980-F19982, F19988, F1999, F550-F554, F558, O99320-O99325
Mental Health Problem/ Any Behavioral Health Diagnosis	Any of the above (depression/dysthymia, PTSD, anxiety, substance abuse) coded = Yes, *or* CME/LE_MentalHealthProblem = Yes	Any codes in the following ranges: 290.0–319.0, 648.3–648.44	Any codes in the following ranges: F0150-F0151, F0280-F0281, F0390-F0391, F04-F99, H9325

^a^Decedent was considered to have the respective condition present if the identified data field had the indicated value recorded

^b^Decedent was considered to have the respective condition present if the identified ICD-9-CM or ICD-10-CM diagnosis codes were assigned during one or more inpatient encounters, or two or more outpatient encounters, within two years of death

### Descriptive variables

Veterans’ demographic and military service characteristics were extracted from VA administrative data. Age was computed at veterans’ dates of death and, for analysis purposes, categorized broadly (due to limited cell sizes within some intent variables) as 18 to 55 or > 55 years. Biological sex was categorized as female versus male (gender data were not available) and marital status as married versus not married. We created two separate indicator variables representing veterans who served in the Vietnam era (yes versus no) or in the post-9/11 era (yes versus no). Veterans’ residential location was categorized as urban versus rural using patients’ addresses and the zip code approximation of the 2010 US urban–rural continuum tables (US Department of Agriculture 2016).

Oregon NVDRS data were used to identify sources of death (categorized as firearm, poisoning, and other) and to categorize the intent of death as suicide, homicide, legal intervention, undetermined or—for firearm-related deaths only—unintentional (no deaths were identified as being due to terrorism). For analysis purposes, we also created a binary variable indicating whether veterans’ deaths were coded as a suicide (yes versus no). Additionally, we examined variables from the Oregon NVDRS indicating whether decedents had a history of treatment, or were currently receiving treatment, for a mental health or substance abuse problem.

### Analyses

Using diagnoses assigned in the VA healthcare system within two years of decedents’ deaths as the criterion standard, we calculated measures of validity for each diagnosis or condition of interest in the Oregon NVDRS, as well as some combinations of diagnoses/conditions (e.g., depression diagnosis *plus current depressed mood*). These measures included overall concordance (i.e., percent agreement), sensitivity, specificity, positive predictive value (PPV), and negative predictive value (NPV). We then examined validity over time and by veterans’ demographic, military service, and death characteristics. Given that veterans may have received healthcare outside of the VA healthcare system, and that we did not have access to non-VA data, this part of the analysis focused only on sensitivity, i.e., the proportion of veterans with a specific VA diagnosis that were correctly coded in Oregon NVDRS data as having the corresponding diagnosis or condition. For each VA behavioral health diagnosis examined, we used logistic regression to calculate odds ratios (ORs) and 95% confidence intervals (CIs) estimating associations between decedents’ characteristics and the accuracy of each variable in the Oregon NVDRS. All data analyses were conducted using SAS software, version 9.3 (SAS Institute, Cary, NC).

## Results

We identified 791 veterans in the Oregon NVDRS data who had used VA healthcare services within the two years prior to death (Table 2). Of these, 92% had received primary care or mental healthcare from the VA; the remainder had used specialty or pharmacy services. Most decedents (95%) were male. Ages of decedents ranged from 20 to 96 years (median = 59) with 60% being 55 or older; 39% were categorized as Vietnam era veterans and 8% as post-9/11 veterans. More than one-third of the decedents were married (39%), and nearly one-half resided in rural/highly rural areas (46%). The majority of deaths were coded as suicides (83%). The remaining deaths were coded as homicides (6%), legal interventions (2%), undetermined intent (8%), or—for firearm-related deaths only—unintentional (0.5%).

**Table 2:** Characteristics of 791 veteran decedents in Oregon NVDRS data, 2003–2017

Characteristic	*n*	%
*Age (years)*		
18–55	317	40.1
> 55	474	59.9
*Sex*		
Male	754	95.3
Female	37	4.7
*Marital Status*^*a*^		
Married	308	39.3
Not Married	475	60.7
*Vietnam Era Veteran*		
Yes	311	39.3
No	480	60.7
*Post-9/11 Veteran*		
Yes	60	7.6
No	731	92.4
*Rurality*^*b*^		
Urban	424	54.3
Rural/Highly rural	357	45.7
*Intent*		
Suicide	656	82.9
Homicide	49	6.2
Legal Intervention	17	2.2
Undetermined	65	8.2
Unintentional (firearm-related only)	*	0.5
*Cause of Death*		
Firearm	487	61.6
Poisoning	156	19.7
Other	148	18.7
*VA Healthcare Diagnoses*		
Depression	286	36.2
PTSD	177	22.4
Anxiety Disorder	130	16.4
Substance Use Disorder	211	26.7
Any Behavioral Health Disorder	473	59.8
*Oregon NVDRS Conditions*		
Depression/Dysthymia Diagnosis	246	31.1
PTSD Diagnosis	99	12.5
Anxiety Disorder Diagnosis	49	6.2
Substance Abuse Problems	223	28.2
Current Depressed Mood	302	38.2
Any Mental Health Problem	335	42.4
*Behavioral Health Treatment*		
History of treatment	309	39.1
Currently receiving treatment	273	34.5
History of/currently receiving treatment	315	39.8

^*^Number is < 10 and is suppressed. ^a^*n* = 8 missing

^b^*n* = 10 missing

*NVDRS* National Violent Death Reporting System; *PTSD* post-traumatic stress disorder

In VA healthcare data, 60% of decedents (*n* = 473) had any behavioral health disorder diagnosis within two years of their death; more than one-third had a depression diagnosis (*n* = 286; 36%), almost one quarter had a PTSD diagnosis (*n* = 177; 22%) or substance use disorder diagnosis (*n* = 211; 27%), and 16% (*n* = 130) had an anxiety disorder diagnosis. In Oregon NVDRS data, 31% of linked decedents (*n* = 246) were documented as having a depression/dysthymia diagnosis, 13% (*n* = 99) as having a PTSD diagnosis, and 6% (*n* = 49) as having an anxiety disorder diagnosis. Documentation of a substance abuse problem, current depressed mood, or a mental health problem was noted in the Oregon NVDRS for 28%, 38%, and 42% (*n* = 223, 302, and 335) of linked decedents, respectively. A substantial proportion of decedents was identified in the Oregon NVDRS data as having a history of (39%), or currently receiving (35%), treatment for a mental health or substance abuse problem (40% were documented in one or both categories).

### Validity of Oregon NVDRS behavioral health variables

Oregon NVDRS-reported behavioral health variables greatly undercounted VA behavioral health diagnoses for depression diagnosis (49% sensitivity), PTSD diagnosis (45% sensitivity), and anxiety disorder diagnosis (17% sensitivity) (Table 3). Although measures of overall concordance were higher (68%, 85%, and 83%, respectively), this was driven primarily by very high measures of specificity (79%, 97%, and 96%), meaning diagnoses were rarely indicated in the NVDRS data in cases where they did not exist in VA data. The ability to accurately identify decedents with VA diagnoses for depression increased when combining the NVDRS variables of depression diagnosis plus current depressed mood (from 49% up to 65% sensitivity); however, this combination resulted in decreased overall concordance (68% to 58%) and specificity (79% to 54%). The NVDRS variable of substance abuse problems identified just over half of decedents who had been diagnosed with a substance use disorder by the VA (56% sensitivity). The agreement between the NVDRS variable for any mental health problem and the VA variable for any behavioral health diagnosis was slightly better than for the individual diagnoses but was also low (59% sensitivity; 82% specificity; 68% concordance). When combining the NVDRS variables of any mental health problem plus substance abuse problems, sensitivity increased (69%) while the specificity decreased (69%) and overall concordance remained nearly the same (69%).

**Table 3:** Validity of Oregon NVDRS behavioral health variables using VA healthcare records as a criterion standard; *n* = 791 veteran decedents, 2003–2017

NVDRS variable	Concordance	Sensitivity	Specificity	PPV	NPV
Depression Diagnosis	0.68	0.49	0.79	0.57	0.73
*plus Current Depressed Mood*	0.58	0.65	0.54	0.45	0.73
PTSD Diagnosis	0.85	0.45	0.97	0.80	0.86
Anxiety Disorder Diagnosis	0.83	0.17	0.96	0.45	0.85
Substance Abuse Problems	0.75	0.56	0.82	0.53	0.84
Any Mental Health Problem	0.68	0.59	0.82	0.83	0.57
*plus Substance Abuse Problems*	0.69	0.69	0.69	0.77	0.60

*NVDRS* National Violent Death Reporting System; *PPV* positive predictive value; *NPV* negative predictive value; *PTSD* post-traumatic stress disorder

### Variations in sensitivity over time and by decedent characteristics

**Fig. 2:**
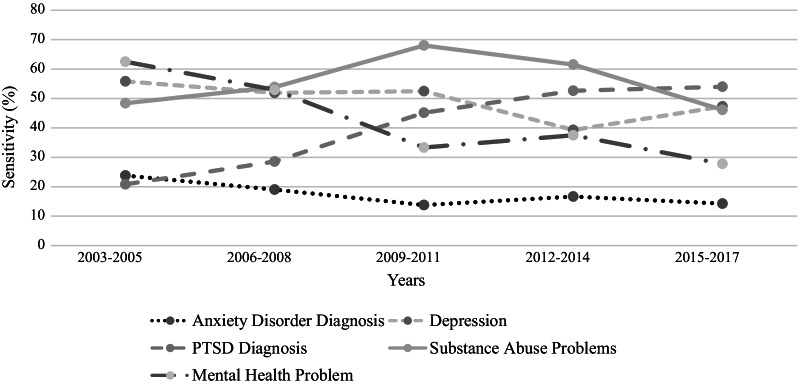
Sensitivity of Behavioral Health Variables in Oregon NVDRS Data by Year, 2003–2017

Sensitivity of Oregon NVDRS behavioral health variables varied over the study period by diagnosis type (Fig. 2). The sensitivity for any mental health problem decreased substantially, from 63% in 2003–2005 to 28% in 2015–2017. In contrast, the sensitivity of the PTSD diagnosis variable increased from 21% in 2003–2005 to 54% in 2015–2017. The sensitivity of the depression and anxiety diagnosis variables remained relatively steady over the study period, though appeared to have an overall downward trend.

Oregon NVDRS data were more likely to accurately identify depression diagnoses for younger versus older veterans (OR = 1.7; 95% CI: 1.1–2.8) and were more likely to accurately identify anxiety diagnoses for those who were married (OR = 3.2; 95% CI: 1.2–8.3) compared to those who were not (Table 4). Oregon NVDRS data were also more likely to correctly identify PTSD diagnoses for decedents identified as post-9/11 veterans (OR = 4.1; 95% CI: 1.8–9.1) compared to veterans from all other service eras; in contrast, they were less likely to correctly identify PTSD diagnoses for Vietnam era veteran decedents (OR = 0.5; 96% CI: 0.3–1.0). No patterns were observed in the validity of NVDRS-coded substance abuse problems relative to VA-diagnosed substance use disorders. However, for any behavioral health diagnosis, Oregon NVDRS data were more likely to accurately identify diagnoses for younger versus older veterans (OR = 1.8; 95% CI: 1.2–2.7) and for post-9/11 veterans versus those from other service eras (OR = 2.6; 95% CI: 1.2–5.6). Conversely, Oregon NVDRS data were less likely to accurately identify any behavioral health disorder diagnoses for those who were married compared to not married (OR = 0.6; 95% CI: 0.4–1.0), those who lived in rural compared to urban areas (OR = 0.6; 95% CI: 0.4–0.9), and those who died by firearm injury compared to those who died from poisoning (OR = 0.4; 95% CI: 0.2–0.6).

**Table 4:** Associations between decedent characteristics and sensitivity of Oregon NVDRS coding of behavioral health diagnoses, by VA healthcare diagnosis type

Characteristic	Depression diagnosis^a^ *n* = 286			PTSD diagnosis^b^ *n* = 177			Anxiety disorder diagnosis *n* = 130			Substance abuse disorder diagnosis *n* = 211			Any behavioral health disorder diagnosis *n* = 473		
	Sensitivity		Bivariable models	Sensitivity		Bivariable models	Sensitivity		Bivariable models	Sensitivity		Bivariable models	Sensitivity		Bivariable models
	*n*	%	OR (95% CI)	*n*	%	OR (95% CI)	*n*	%	OR (95% CI)	*n*	%	OR (95% CI)	*n*	%	OR (95% CI)
*Age (years)*															
18–55	85	55.6	**1.7 (1.1–2.8)**	51	48.1	1.4 (0.8–2.6)	11	15.1	0.7 (0.3–1.9)	75	57.7	1.2 (0.7–2.1)	183	75.3	**1.8 (1.2–2.7)**
> 55	55	41.4	–	28	39.4	–	11	19.3	–	43	53.1	–	145	63.0	–
*Sex*															
Female	14	56.0	1.4 (0.6–3.1)	*	38.9	0.8 (0.3–2.1)	*	30.0	2.3 (0.5–9.6)	*	81.8	3.8 (0.8–17.8)	23	76.7	1.5 (0.6–3.5)
Male	126	48.3	–	72	45.3	–	19	15.8	–	109	54.5	–	305	68.8	–
*Marital Status*															
Married	50	45.5	0.8 (0.5–1.3)	35	48.6	1.3 (0.7–2.4)	12	27.3	**3.2 (1.2–8.3)**	32	46.4	0.6 (0.3–1.0)	114	63.7	**0.6 (0.4–1.0)**
Not Married	90	51.1	–	44	41.9	–	*	10.6	–	86	60.6	–	213	72.7	–
Vietnam Era Veteran															
Yes	58	49.2	1.0 (0.6–1.6)	27	35.5	**0.5 (0.3–1.0)**	*	22.0	1.6 (0.6–4.2)	55	59.1	1.3 (0.7–2.2)	136	68.7	0.9 (0.6–1.4)
No	82	48.8	–	52	51.5	–	13	14.6	–	63	53.4	–	192	69.8	–
*Post-9/11 Veteran*															
Yes	17	47.2	0.9 (0.5–1.9)	25	71.4	**4.1 (1.8–9.1)**	*	13.6	0.7 (0.2–2.8)	16	59.3	1.2 (0.5–2.7)	43	84.3	**2.6 (1.2–5.6)**
No	123	49.2	–	54	38.0	–	19	17.6	–	102	55.4	–	285	67.5	–
*Rurality*^*a*^															
Urban	83	48.8	–	47	44.3	–	15	17.9	–	72	59.0	–	196	74.2	–
Rural	56	49.6	1.0 (0.6–1.7)	32	46.4	1.1 (0.6–2.0)	*	15.9	0.9 (0.3–2.3)	44	52.4	0.8 (0.4–1.3)	130	64.0	**0.6 (0.4–0.9)**
*Suicide*															
Yes	119	50.9	1.5 (0.8–2.8)	62	45.3	1.1 (0.5–2.3)	15	14.2	0.4 (0.1–1.1)	92	56.1	1.0 (0.5–2.0)	270	70.1	1.2 (0.7–2.0)
No	21	40.4	–	17	42.5	–	*	29.2	–	26	55.3	–	58	65.9	–
*Mechanism of Death*															
Firearm	66	42.3	0.6 (0.3–1.0)	44	44.4	1.1 (0.6–2.3)	*	12.7	0.5 (0.1–1.3)	57	55.9	0.9 (0.5–1.8)	160	61.8	**0.4 (0.2–0.6)**
Poisoning	45	55.6	–	21	41.2	–	*	23.7	–	32	58.2	–	91	82.0	–
Other	29	59.2	1.2 (0.6–2.4)	14	51.9	1.5 (0.6–3.9)	*	17.2	0.7 (0.2–2.3)	29	53.7	0.8 (0.4–1.8)	77	74.8	0.7 (0.3–1.3)

^*^Numbers are < 10 and are suppressed

Bold font indicates statistical significance at *p* < 0.05

^a^Rurality data missing for *n* = 3 with depression diagnosis, *n* = 2 with PTSD diagnosis, *n* = 5 with anxiety diagnosis, *n* = 2 with substance use disorder diagnosis, and *n* = 6 for any behavioral health disorder diagnosis

^b^Marital status data missing for *n* = 1 with anxiety diagnosis and *n* = 1 with any behavioral health disorder diagnosis

*CI* confidence interval; *NVDRS* National Violent Death Reporting System; *OR* odds ratio; *PTSD* post-traumatic stress disorder

Note: “–" indicates the reference group for each comparison

## Discussion

To our knowledge, this is the first study to evaluate potential misclassification of behavioral health-related variables collected in the NVDRS compared to a healthcare record-based criterion standard. Our results suggest that mental health and substance use problems may be severely undercounted among decedents and that the validity of some of these variables may vary systematically by decedent characteristics. Understanding these patterns can inform future studies that leverage these important data while also pointing toward potentially effective quality improvement efforts. This work is timely given the recent expansion of this important surveillance system to all states and several territories in the US along with increasing efforts to utilize the data for public health practice (e.g., Barber et al. 2019; Nazarov et al. 2019; Ranade et al. 2020).

Given that we had access to decedents’ healthcare records from only one healthcare system, we focused our analyses on the *sensitivity* of behavioral health indicators in the NVDRS – in other words, the likelihood that NVDRS data accurately reported the presence of a behavioral health condition identified in decedents’ VA healthcare records. We did not focus as much on other measures of accuracy such as specificity, PPV, NPV, or overall concordance because of the possibility that veterans received care for their behavioral health conditions outside the VA healthcare system (Hynes et al. 2007; Gellad 2016). It is also possible that decedents’ behavioral health conditions are either not diagnosed or, if diagnosed, simply not detected by NVDRS abstractors using the available data systems. It is notable that, for most conditions examined, the specificity of NVDRS variables—and overall concordance (percent agreement), which is a function of both sensitivity and specificity—was high, meaning that decedents did not tend to be coded as having a condition where their VA healthcare record did not include a diagnosis for that condition. This finding suggests that NVDRS abstractors are not coding conditions unless clear evidence of a diagnosis exists, as instructed in the NVDRS coding manual (US Centers for Disease Control and Prevention 2021). Our results showing higher sensitivity when combining variables that do not require as clear of evidence—e.g., current depressed mood or substance abuse problems—also supports this case.

It is noteworthy that 40% of decedents had documentation of receiving current or past treatment for behavioral health problems, yet there was still considerable underreporting of the conditions themselves. This may be a result of less stringent criteria used for the treatment variables relative to the variables reflecting the individual conditions. It also seems likely that the systematic underreporting of behavioral health conditions is coming from incomplete information in the sources of data used for NVDRS abstraction—another reminder of the imperfect nature of NVDRS variables in cases where investigators seek to measure actual clinical diagnoses. Knowledge of this misclassification, and its potential causes, is important when NVDRS data users interpret their results and apply findings to prevention efforts. Future work that further examines the reasons for underreporting of behavioral health conditions will help elucidate mechanisms for improvement.

Although our analysis was specific to veterans who used VA healthcare, it is unlikely that the observed undercounts are unique to veteran decedents or to VA healthcare users. Indeed, past research has shown that, in cases of suicide, death reports undercounted cases of major depression and substance abuse relative to the results of full psychological autopsies (Draper et al. 2007). As such, more routine access to healthcare records by those conducting death investigations would likely improve our national surveillance system of violent deaths. Whether this is feasible, however, is unknown; in the US, the extent of a death investigation, including interviews or acquisition of healthcare records, is highly variable across jurisdictions and, due to resource limitations, is often not completed for all referred cases (Hickman et al. 2004; US Department of Justice 2019). This differential access to resources may help explain the statistically significant decrease in sensitivity of NVDRS data to identify behavioral health diagnoses among rural versus urban decedents. Additionally, the fragmented nature of the US healthcare system—making it likely that investigators would need to retrieve records from multiple systems—adds additional complications to acquiring healthcare records. Alternate sources of information on decedents’ behavioral health conditions are likely needed in order to utilize death records and, relatedly, NVDRS data to examine and compare behavioral health issues among decedents.

In our sample, the vast majority (83%) of veteran deaths were due to suicide among men, and firearms were involved in nearly two-thirds (61%) of deaths. These proportions are known to be higher among veteran decedents than among non-veteran decedents (Kaplan et al. 2009) and, as such, their magnitude in our sample was not surprising. Due to heightened awareness of veteran suicide, we expected that deaths identified as suicides would have systematically higher sensitivity in behavioral health coding than non-suicide deaths; however, no patterns by cause of death were identified. By *mechanism* of death, there was an increased likelihood that those due to poisoning, compared to those associated with firearms, correctly identified veterans with any mental health disorder diagnosis. It may be that poisoning cases are more thoroughly investigated—for example, if there is a need to examine decedents’ sources of lethal prescription medications. On the other hand, it is also important to consider whether the lower sensitivity among deaths associated with firearms is due to stigma related to this mechanism of death, to more nuanced or alternative behavioral health issues than those examined in this study, or to other related issues. Future work that examines these factors could further elucidate methods to improve data collection for all decedents captured in the NVDRS. Additional investigation into completeness of death certificate or vital records data, and consistency (e.g., interrater reliability) across NVDRS abstractors, might also highlight areas for quality improvement.

The sensitivity of PTSD coding in the NVDRS data appeared to increase substantially over the years examined. Somewhat relatedly, it appeared that PTSD was more accurately identified for the newest era of veterans who served post-9/11, especially in contrast to Vietnam era veterans, for whom VA-diagnosed PTSD was much less likely to be accurately identified. These patterns may be explained by the fact that the US was at war for most of the existence of the NVDRS, and that awareness of the deleterious effects and need for treatment of PTSD among this newest generation of combatants was higher than among previous generations of veterans (Goldberg et al. 2019). It may also be that more thorough investigations are completed for young decedents than for older decedents. Our finding that both the depression and mental health problem variables had greater sensitivity for younger decedents than for older decedents supports this possibility.

Some strengths and limitations of this study design should be taken into consideration when interpreting results. Strengths include our ability to link decedents’ records to an entire healthcare system and to restrict analyses to those with recent healthcare visits. This is a unique use of linked data that, to our knowledge, has not been previously utilized to examine NVDRS validity. However, our analysis is limited by our focus on only a single state and healthcare system. As such, data may have excluded a small number of Oregon decedents who died out-of-state. Further, these findings may not represent patterns in other states’ NVDRS systems or among all decedents, an especially notable limitation given the broad variability across states in medicolegal investigations (Oregon being among the states with a centralized medical examiner system; Hickman et al. 2004; Ruiz et al. 2018; US Department of Justice 2019). Our analyses were also limited by a relatively small numbers of observations for analysis, making some of the estimated ORs unstable (i.e., wide confidence intervals) in our examination of patterns of sensitivity by decedent characteristics. Future work that replicates this analysis across a larger number of states and with diagnosis data from additional healthcare systems will yield greater statistical power and suggest whether our findings are localized to Oregon and to veterans or are generalizable to the system as a whole.

## Conclusions

Linking data between the VA healthcare system and Oregon’s NVDRS database allowed us to estimate the validity of a single state’s NVDRS behavioral health variables relative to decedents’ actual healthcare diagnoses. We observed low sensitivity of these variables compared to diagnoses made within two years of death, suggesting that healthcare records are not systematically queried in a way that would provide accurate behavioral health information. We also observed some systematic differences in sensitivity by veterans’ demographic characteristics, and by intent and date of death. The NVDRS is a critically important, national surveillance system allowing public health professionals to examine patterns of, and risk factors for, violent death in the US. Efforts to examine the validity of behavioral health variables for non-veteran decedents, and in states other than Oregon, would further our understanding of these gaps and point the way to fruitful quality improvement efforts.

## Data Availability

The datasets used and/or analyzed during the current study are available from the corresponding author on reasonable request.

## Abbreviations

CDC: Centers for disease control and prevention
CI: Confidence interval
ICD-9-CM: International classification of diseases–9th revision–clinical modification
ICD-10-CM: International classification of diseases–10th revision–clinical modification
NPV: Negative predictive value
NVDRS: National violent death reporting system
OR: Odds ratio
PPV: Positive predictive value
PTSD: Post-traumatic stress disorder
US: United States
VA: Department of Veterans Affairs
